# Nerve Cross-Bridging to Enhance Nerve Regeneration in a Rat Model of Delayed Nerve Repair

**DOI:** 10.1371/journal.pone.0127397

**Published:** 2015-05-27

**Authors:** Tessa Gordon, Michael Hendry, Christine A. Lafontaine, Holliday Cartar, Jennifer J. Zhang, Gregory H. Borschel

**Affiliations:** Department of Surgery, Hospital for Sick Children, University of Toronto, Toronto, Ontario, Canada; University of Sydney, AUSTRALIA

## Abstract

There are currently no available options to promote nerve regeneration through chronically denervated distal nerve stumps. Here we used a rat model of delayed nerve repair asking of prior insertion of side-to-side cross-bridges between a donor tibial (TIB) nerve and a recipient denervated common peroneal (CP) nerve stump ameliorates poor nerve regeneration. First, numbers of retrogradely-labelled TIB neurons that grew axons into the nerve stump within three months, increased with the size of the perineurial windows opened in the TIB and CP nerves. Equal numbers of donor TIB axons regenerated into CP stumps either side of the cross-bridges, not being affected by target neurotrophic effects, or by removing the perineurium to insert 5-9 cross-bridges. Second, CP nerve stumps were coapted three months after inserting 0-9 cross-bridges and the number of 1) CP neurons that regenerated their axons within three months or 2) CP motor nerves that reinnervated the extensor digitorum longus (EDL) muscle within five months was determined by counting and motor unit number estimation (MUNE), respectively. We found that three but not more cross-bridges promoted the regeneration of axons and reinnervation of EDL muscle by *all* the CP motoneurons as compared to only 33% regenerating their axons when no cross-bridges were inserted. The same 3-fold increase in sensory nerve regeneration was found. In conclusion, side-to-side cross-bridges ameliorate poor regeneration after delayed nerve repair possibly by sustaining the growth-permissive state of denervated nerve stumps. Such autografts may be used in human repair surgery to improve outcomes after unavoidable delays.

## Introduction

Despite optimal management, recovery of function after peripheral nerve injury and surgical repair is rarely complete; indeed, it is frequently poor [1,2]. Whilst this is usually attributed to fat replacement of atrophic denervated muscles [2], the progressive failure of nerve regeneration over time and distance is accounted for by a progressive decline in the regenerative capacity of the injured neurons and the diminished regenerative support by the chronically denervated Schwann cells within the denervated nerve stumps [3–9]. The declining regenerative capacity is accompanied by a corresponding decline in the expression of growth-associated genes [3–9].

Chronically denervated muscles contract more forcefully after delayed nerve repair when a prior surgery was carried out to direct sensory nerves into the denervated muscle [10]. The explanation given for the findings was that the sensory nerves ‘protected’ the muscles [10]. Axon-mediated “Schwann cell protection” was indicated as a contributing factor [11]. We recently re-examined this issue with the surgical procedure of two end-to-side neurorrhaphies which does not require the sacrifice of any nerve. The surgical technique is to place nerve autografts at right angles between a donor intact nerve and a recipient denervated nerve stump with the autografts each connected in an end-to-side manner [12]. Viterbo and colleagues pioneered the technique with electrophysiological and histological evidence that axons passed through the autografts [13]. They did not, however, examine how many axons passed through, their destination(s), nor the outcomes of the procedure. The technique, referred to as side-to-side cross-bridging by Ladak et al [12], is an extension of the more widely used end-to-side nerve coaptation (end-to-side neurorrhaphy) of a recipient denervated distal nerve stump into the side of a donor intact nerve, with and without a perineurial window [14–21]. Retrograde tracing used in the later study of Ladak et al revealed that ~50 tibial motoneurons sent their axons across three cross-bridges into a recipient denervated common peroneal (CP) distal nerve stump. The tibial axons ‘protected’ chronically denervated Schwann cells because the number of motoneurons that regenerated their axons after delayed coaptation of the proximal and distal CP nerve stumps was increased 1.7-fold [12].

There remain many unanswered and important questions. These are addressed in the current study: 1) Does the size of perineurial windows cut into a donor nerve and a recipient denervated nerve stump impact on the number of neurons that grow axons through side-to-side cross-bridges? 2) Do donor axons that enter the denervated distal nerve stump through side-to-side cross-bridges, continue to grow either proximal and/or distal to the cross-bridges, possibly influenced by a neurotrophic effect of the denervated targets? 3) Do both motor *and* sensory neurons contribute their axons through the side-to-side cross-bridges? 4) Does the number of cross-bridges dictate how many neurons participate in growing axons through the cross-bridges? and 5) Will optimum placement of side-to-side cross-bridges improve the regeneration of motor nerves and/or sensory nerves after delayed nerve repair? In light of there being no reliable medical or surgical options to oppose poor functional recovery when nerves regenerate through chronically denervated Schwann cells, our objective is to promote nerve regeneration and muscle reinnervation after delayed repair by ‘protecting’ chronically denervated nerve stumps with axons from a nearby donor nerve.

## Materials and Methods

### Animals

All experiments were carried out on adult female Sprague Dawley rats (250-270g; n = 110). Of these, 26 rats were *Thy-*1-GFP transgenic rats that were developed in our laboratory. Green fluorescent protein (GFP) is expressed in neural cells under the Thy-1 promoter but is not expressed in muscle or connective tissue [22–24].

### Surgery

Sterile surgery was carried out under inhalational anesthetic (2% Isofluorane in 98% oxygen; Halocarbon Laboratories, River Edge, NJ) and using an operating microscope (Leitz, Willowdale, ON). The rats were administered subcutaneous Metacam (0.3 mL/100 g body weight; Boehringer Ingelheim Vetmedica Inc., St. Joseph, MO) for relief of post-operative pain. A warming pad was placed under the rat in order to maintain body temperature during surgery and during the brief period during which the rat regained consciousness after the surgery when the inhalational anesthetic administration ceased. The protocol was approved by the Animal Care Committee, Hospital for Sick Children, Toronto, and all perioperative care, including postoperative monitoring and analgesia, was administered according to Canadian Council for Animal Care guidelines. Post-operative care included regular daily post-operative monitoring over the first week of all surgeries followed by regular monitoring by the staff of the animal facility.

**Fig 1:**
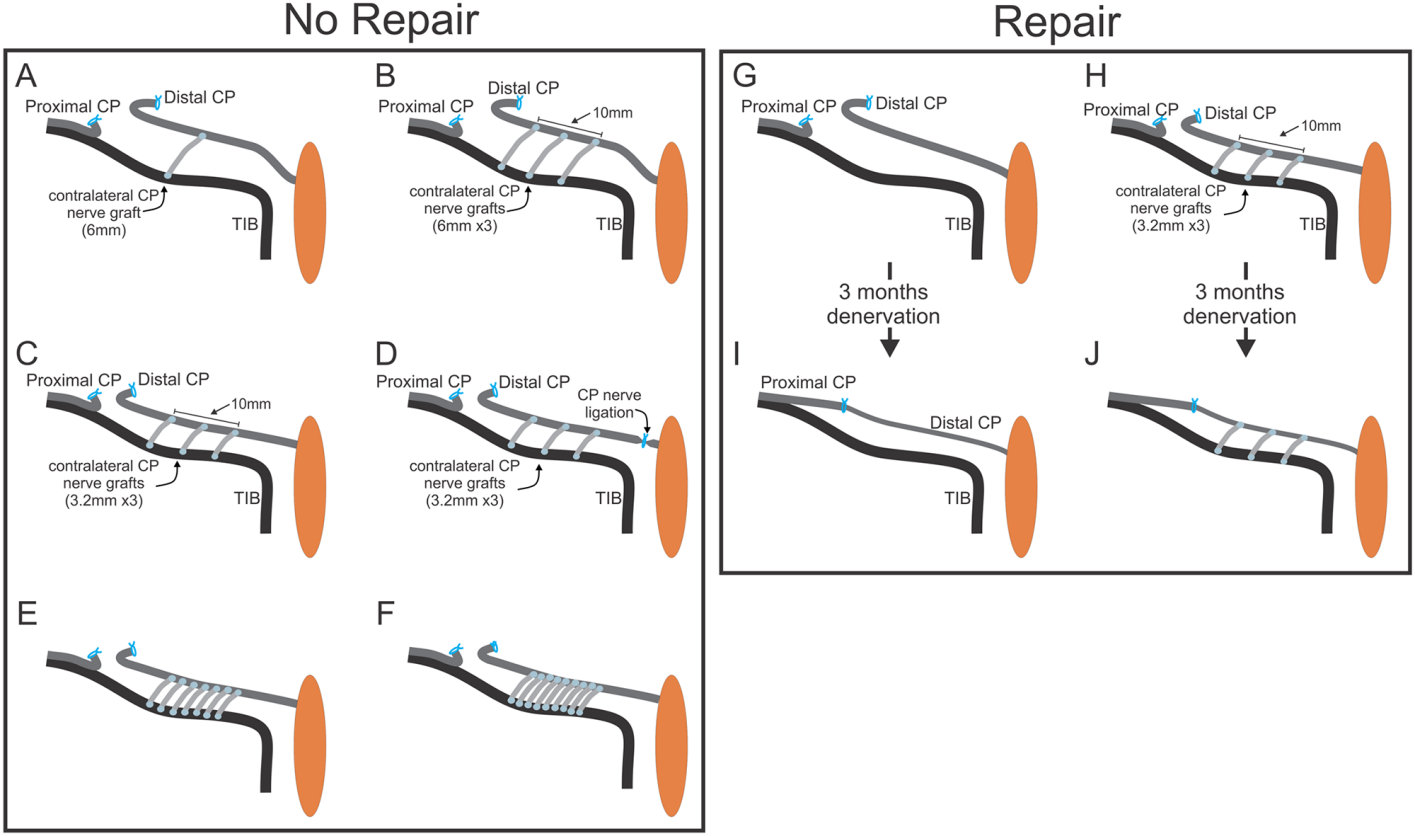
The surgical placement of autografts as side-to-side cross-bridges between an intact donor tibial (TIB) and a recipient denervated common peroneal (CP) distal nerve stump and the delayed coaptation of the CP nerve stumps. In the first set of experiments (No Repair: Table 1), the distal nerve stump of transected CP nerve was laid parallel to the intact TIB donor nerve for securing CP autologous cross-bridges at right angles to the nerves: 1 and 3 (5 and 7 not shown) cross-bridges 6 mm long (A,B) or 3 to 9 cross-bridges 3.2 mm in length (C-F). Perineurial windows were opened in the TIB and CP nerves to insert 1–3 bridges over a distance of 10 mm on each nerve (A-C). To examine possible neurotrophic influence of the denervated targets, the connection of the denervated CP nerve with these targets, the nerve was ligated (D). To insert more than 3 bridges of 3.2 mm length, the perineurium was removed (E-F). In a second set of experiments (Repair, Table 1), no cross-bridges were placed between the donor TIB nerve and recipient denervated CP nerve stump in a control group of rats (G) and 3–9 CP autologous cross-bridges were placed between the nerves in the experimental rat group (H) (only 3 cross-bridges shown). Three months later, the CP nerve stumps were refreshed and sutured together to encourage CP nerve regeneration through either an ‘unprotected’ chronically denervated CP nerve stump where no cross-bridges were placed (I) or through a CP nerve stump that was ‘protected’ by placing 3–9 cross-bridges between the donor TIB nerve and the denervated CP nerve stump (J).

In the first experiment (Experiment #1; N = 68 rats; Table 1), side-to-side autologous common peroneal (CP) cross-bridges were inserted between a donor intact tibial (TIB) nerve and a recipient denervated CP nerve stump. CP and TIB nerves were exposed in both hindlimbs through gluteal muscle-splitting incisions. The *left* CP nerve was dissected ~5–7 mm from the sciatic notch to its distal insertion into the muscles in the anterior hindlimb compartment. The CP nerve was carefully dissected free from the TIB nerve along the natural division of the two nerves as they course through the sciatic nerve. Dissection was halted at the most proximal portion of the sciatic nerve at the sciatic notch where the CP, TIB, and sural nerve fascicles were interweaved. A ~28–30 mm nerve length was harvested, cut, and kept moist in the hindlimb for later division into one to nine autologous CP side-to-side nerve cross-bridges. In the *right* experimental hindlimb, the CP nerve was transected and the proximal and distal nerve stumps were ligated. The distal CP nerve stump was laid parallel to the donor TIB nerve and the two CP nerve stumps were sutured to underlying innervated muscle to prevent nerve regeneration from the proximal to the distal nerve stump [25]. CP cross-bridges from the *left* hindlimb were inserted into the sides of the donor TIB nerve and the recipient denervated CP nerve stump through equidistant perineurial windows placed over a 10 mm length (Fig 1A–1D). The radius of the windows was increased from ~125 to 500 μm (Table 1).

**Table 1:** The two sets of experiments (Exp.) in which 1–9 cross-bridges were inserted through perineural windows between the donor tibial nerve and the recipient common peroneal (CP) nerve in Exp 1, and denervated common peroneal (CP) nerve stumps were coapted three months after placing the cross-bridges in Exp. 2.

Experiment sets and Rat numbers		Number of side-to-side cross-bridges						Perineurial window radius and bridge length (mm)	
Experiments: Q & A	Rats	0	1	3	5	7	9	radius	length
**Exp. 1A**									
Does window size affect axon crossing? *Yes*, *with most axons regenerating rather than sprouting*	20	-	-	7	5	8	-	0.125	6
	12	-	6	6	-	-	-	0.250	6
**Exp. 1B**									
Do donor axons grow in either direction in the recipient nerve stump? *Yes*, *even when disconnected from denervated targets*	12	-	-	12	-	-	-	0.50	3.2
	6			6					
**Exp. 1C**									
Do more donor axons grow through bridges when window size is increased to incorporate >3 bridges? *No they do not*.	18	-	-	-	6	6	6	10.00	3.2
**Exp. 2A**									
Does the ‘protection’ by 3 cross-bridges improve nerve regeneration and muscle reinnervation after delayed repair? *Yes*, *increasing the regeneration of both motor and sensory axons 3-fold as well as increasing muscle reinnervation*	12	-	-	12	-	-	-	0.50	3.2
	12	12	-	-	-	-	-	0	3.2
**Exp 2B**									
Does ‘protection’ by >3 bridges improve this regeneration? *No*, *>3 bridges did not improve regeneration*	18	-	-	-	6	6	6	10.00	3.2

The research questions addressed are shown with the answers in italics.

The windows were made by lifting the perineurium with a fine forceps to open and extend the incision. Their size was varied to insert cross-bridges in the following configuration: three, five, or seven 6 mm long bridges were inserted through perineurial windows (~125 μm radius, n = 20), one and three 6 mm long bridges (~250 μm radius, n = 12; Fig 1A and 1B), three 3.2 mm long bridges (~500 μm radius, n = 12; Fig 1C) and listed under Exp.1B in Table 1. To test for possible target neurotrophism, the distal end of the recipient denervated CP nerve stump was ligated in rats in which 3.2 mm long bridges were inserted through perineurial windows of a ~500 μm radius (n = 6; Fig 1D) as listed under Exp.1B in Table 1. When more than three bridges were placed, the perineurium was removed over the 10 mm length of the donor and recipient nerves. We placed five, seven, and nine 3.2 mm long cross-bridges after removing the perineurium (n = 18; Fig 1E and 1F) as listed under Exp 1C in Table 1. The autologous CP nerve cross-bridges were secured at right angles to the donor TIB nerve and the recipient denervated CP nerve stump by applying Tisseel glue (Baxter, Mississauga, Ontario; 1:1 mix of thrombin and fibrinogen). Thereafter the internal and external incisions were closed with 5–0 silk and 4–0 Vicryl suture, respectively.

**Fig 2:**
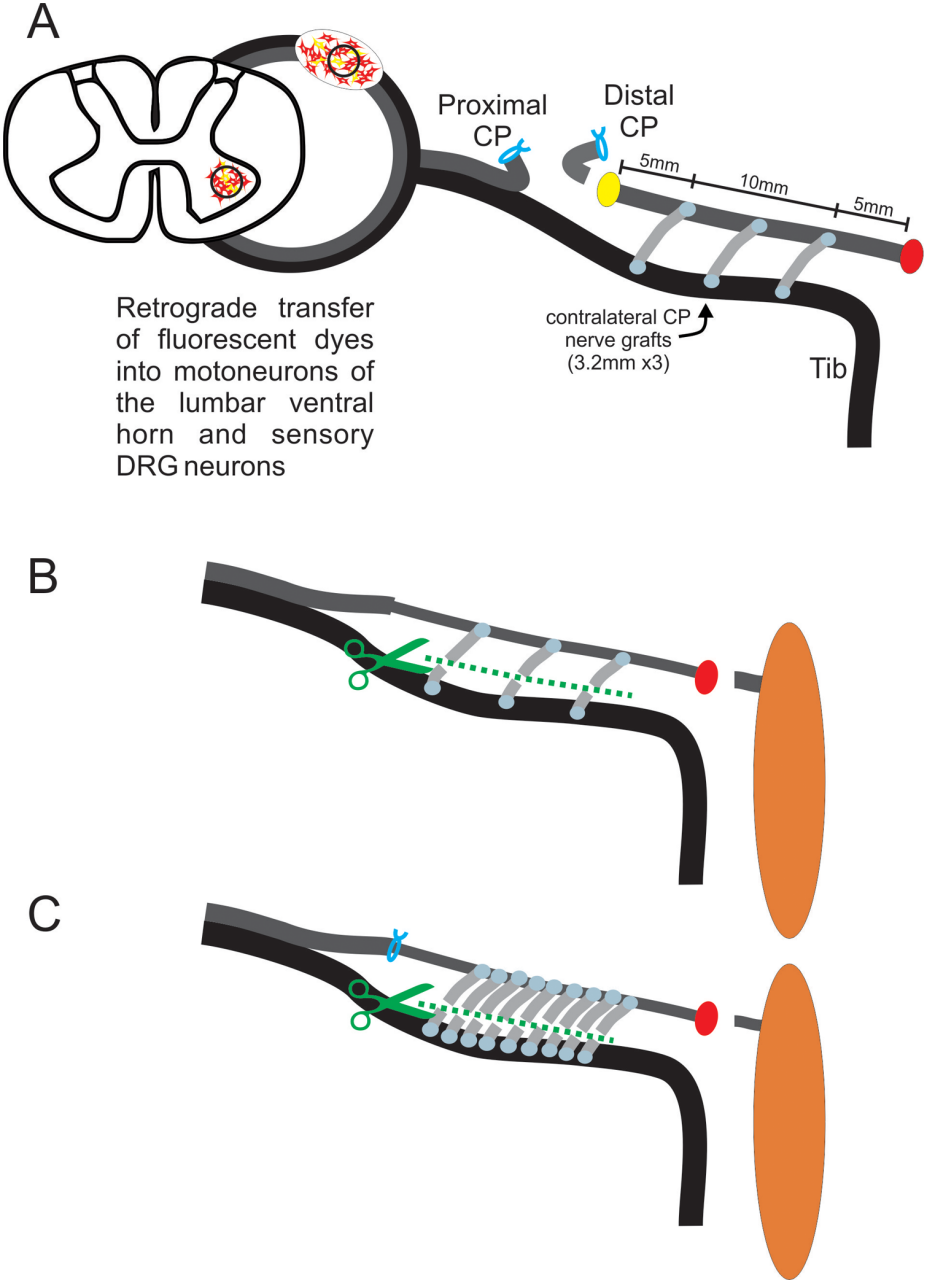
Retrograde labeling of motor and sensory tibial (TIB) neurons that grow axons from a donor TIB nerve into a recipient denervated common peroneal (CP) nerve and, of CP neurons that regenerate their axons after delayed CP nerve repair. In the first set of experiments (No Repair: Table 1) A. Fluorogold (FG) and fluororuby (FR) retrograde dyes were applied to the CP nerve stump 5 mm either side of 3.2 mm long cross-bridges in order to identify the TIB neurons that had grown axons through the cross-bridges into the recipient denervated CP nerve stump. B,C. In a second set of experiments (Repair, Table 1), 5 months after delayed repair of CP nerve, FG or FR was applied to the regenerated CP axons to enumerate the CP neurons that had regenerated through the denervated CP nerve stump ‘protected’ by 3 (B) and 9 (C) bridges, as examples. By cutting through the cross-bridges at the same time as the dye application, retrograde labelling was confined to only the CP neurons that had regenerated their axons through the cross-bridges (B,C).

In a second sterile surgery carried out three months later in the right experimental hindlimb, donor TIB neurons that had grown their axons into the recipient denervated CP nerve stump either side or both sides of the inserted cross-bridges were retrogradely labeled. In the rats in which 3.2 mm cross-bridges were placed (Fig 2A) as listed under Exp. 1B and C in Table 1, a 2–3 mm long CP nerve sample was taken for morphology (see below) prior to exposing the axons of the TIB neurons to a 4% solution of fluorogold (FG; Hydrostilbamidine bis(methanesulphonate), Sigma #39286) or an 8% solution of fluororuby dextran tetramethylrhodamine (FR; Molecular Probes, Eugene, OR, USA) solution. The transected tips of the donor TIB nerve and the CP nerve stump were exposed to the dyes within Vaseline wells for one hour, the dyes being randomized to the CP nerve stump either side of the cross-bridges. Thereafter the wells were removed and the exposed nerves checked for visible staining. The incision site was then closed and the rats were allowed to recover.

**Fig 3:**
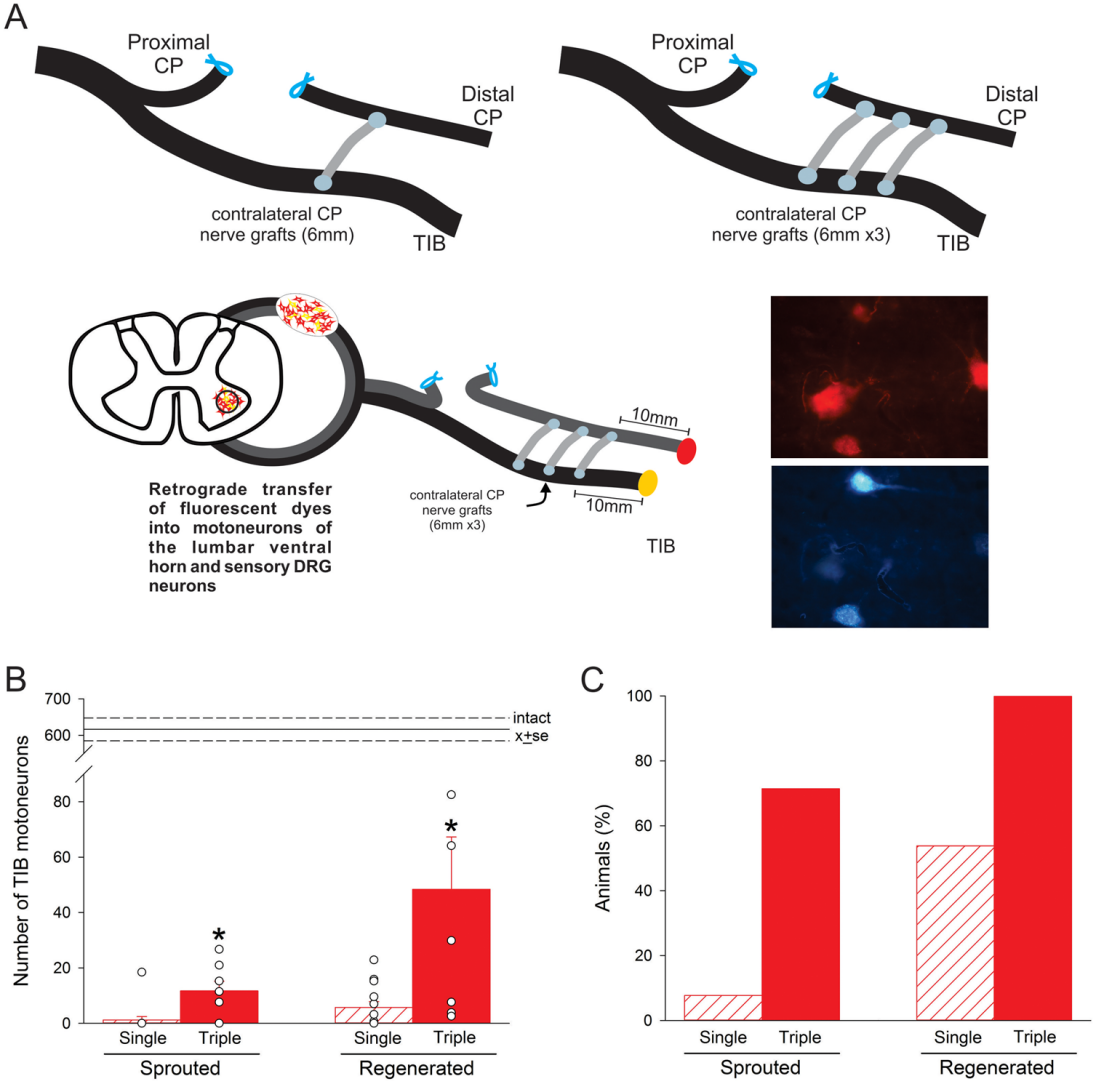
More donor tibial (TIB) motoneurons regenerate axons into a recipient common peroneal (CP) nerve stump when 3 rather than a single cross-bridge was secured between perineurial windows in the nerves; few motoneurons sprout axons through the cross-bridges. In the first set of experiments (No Repair: Table 1), A. 1 or 3 autologous CP nerve cross-bridges (6 mm long) were placed between a donor TIB nerve and a recipient denervated CP nerve stump via perineurial windows (with a radius of~250 μm) that were opened in both nerves. Three months later, fluorogold (FG) and fluororuby (FR) retrograde dyes were applied to the CP and TIB nerves distal to the cross-bridges in order to identify TIB motoneurons that had sprouted (containing *both* dyes) and those that had regenerated (containing only FR) axons into the recipient denervated CP distal nerve stump. B. Mean ± SE of the numbers of TIB motoneurons that sprouted and regenerated their axons into the recipient denervated CP nerve stump (data points for individual rats are shown) and C. the proportion of rats (as a percentage) in which sprouted and regenerated TIB motoneurons were found. In B. significant differences are shown by the * with more neurons sprouting *and* regenerating axons through 3 cross-bridges as compared to 1 cross-bridge.

In the rats in which one or three 6 mm long CP autografts were placed (Fig 3A and 3B), the recipient CP distal nerve stump was exposed to one dye 10 mm distal to the cross-bridges in order to backlabel TIB neurons that sent axons into the denervated CP distal nerve stump. The donor TIB nerve was also cut 10 mm distal to the location of the cross-bridge placement for retrograde labeling with the second dye (Fig 3A). In this manner, those TIB neurons that were backlabelled with the dye applied to the recipient CP nerve alone were identified as the TIB neurons that had regenerated their axons across the bridges and into the recipient denervated CP nerve stump. Typical examples are the two middle motoneurons in the photomicrograph in Fig 3A that contain only the FR dye. Those TIB neurons that contained both dyes were neurons that had sprouted axons through the cross-bridges, retaining their axons within the donor TIB nerve distal to the cross-bridges (Fig 3A). In the same manner as for the first group of rats where donor TIB neurons that had grown their axons either side of the cross-bridges into the recipient denervated nerve stump, backlabeling was performed by exposing the transected tips of the donor TIB nerve and the CP nerve stump to the dyes for one hour. One week after retrograde labelling, rats were transcardially perfused with 4% paraformaldehyde under deep isofluorane anesthesia. The lumbosacral spinal cord and the dorsal root ganglia (DRG) at the L4-5 levels that contained the CP and TIB motor and sensory neuron pools, respectively, were surgically removed and embedded in optimum cutting temperature medium (OCT). In the right experimental hindlimb of the *Thy-1* GFP transgenic rats, the donor TIB nerve and the recipient denervated CP nerve stump that were ‘bridged’ by side-to-side CP nerve autografts were dissected, the extraneous connective tissue carefully removed, and the nerves embedded longitudinally in OCT and frozen at -20°C.

In a second experiment (Experiment #2; Table 1), autologous CP nerve cross-bridges were dissected from the left hindlimb and placed into the right hindlimb in a second group of 30 rats (Fig 1H) under the same aseptic conditions and using the same drugs for anesthesia and pain management. A control group of rats (n = 12) was included in which the same CP transection and ligation of the proximal and distal stumps was carried out in the right experimental hindlimb but no autologous CP cross-bridges were placed (Fig 1G) as listed in Exp. 2A in Table 1. In the second sterile surgery carried out three months later, the ligated CP nerve stumps in the experimental right hindlimb were identified and examined visually. Thereafter, the proximal nerve stump was electrically stimulated via bipolar electrodes to ensure that no axon regeneration had occurred between the ligated CP nerve stumps over the three month period. The proximal and distal CP nerve stumps were then freed and refreshed for microsurgical coaptation (Fig 1I and 1J), using 10–0 silk thread within a ~2.5 mm long Silastic tube, as described in detail previously [25]. Briefly, the two CP nerve stumps were opposed by inserting a needle attached to the 10–0 silk thread through the center of the tube, through the perineurium of the proximal and then the distal nerve stump, and exiting the tube at the same site. The two stumps of the transected CP nerve were gently pulled together by pulling the silk suture, the apposition of the nerve stumps being aided by capillary suction within the silastic tube [25]. The suture was tied, the incisions closed, and the rats allowed to recover on a heat pad.

In a third aseptic surgery performed three months after CP microsurgical coaptation, CP neurons that had regenerated their axons across the coaptation site and beyond the cross-bridges were retrogradely with either FG or FR dyes (Fig 2B and 2C). The dye was applied to the CP distal nerve stump for one hour, 10 mm from the most distally placed side-to-side cross-bridge and ~25 mm from the CP coaptation site. In the rats in which cross-bridges had been placed, the bridges were cut to ensure that *only* the CP neurons that had regenerated their axons through the distal CP nerve stump were backlabelled and *not* the donor TIB neurons whose axons had grown through into the denervated CP distal nerve stumps prior to the coaptation of the CP nerve stumps (Fig 2B and 2C). In the control group of rats in which no bridges were placed, the same location on the distal nerve stump was cut to apply the retrograde dyes. Surgical closure of the wound followed and the rats were allowed to recover on a heat pad. One week later, transcardial perfusion was carried out as described for Experiment #1 for dissection and removal of the lumbosacral spinal cord and the DRGs.

### Data collection

#### Neuronal and axon counts

Fifty and 20 μm thick sagittal sections of the lumbosacral spinal cord and the DRGs, respectively, were cryosectioned for visualization and counting of 1) the donor TIB motor and sensory neurons that regenerated and/or sprouted their axons into the recipient denervated CP nerve stump in the right experimental hindlimb (Exp. 2A-C in Table 1), and 2) CP motor and sensory neurons that regenerated their axons into the chronically denervated CP nerve stump which had and had not been ‘protected’ by autologous CP side-to-side cross-bridges prior to delayed CP nerve repair (Exp. 2A,B in Table 1), again in the right experimental hindlimb. The sections were visualized and the retrograde labeled neurons counted under a fluorescent filter with an excitation band pass of 470–490 nm for FG and of 580–620 for FR dyes. All the backlabelled motoneurons were counted in every spinal cord section whilst DRG sensory neurons were visualized and counted on every 5^th^ section. Only those neurons in which a distinct nucleus was visible were counted. The numbers were corrected to control for the counting of split nuclei using the method of Abercrombie [26].

A 2–3 mm length of CP nerve was excised for histomorphometry prior to backlabelling the TIB neurons in the second sterile surgery of the second experiment (Exp. 2A and B in Table 1). The nerve was fixed overnight in 2.5% gluteraldehyde solution buffered in 0.025 M cacodylate, washed, and then stored in 0.15 M cacodylate buffer. Samples were subsequently post-fixed in 2% osmium tetroxide, dehydrated in a series of graded alcohols, and embedded in EPON. Nerve cross-sections of 0.6 μm were cut through the center of the sample and stained with toluidine blue for viewing at the light microscopic level. Entire nerve cross-sections were captured at 1000x magnification (Leica DM2500) and the myelinated axons were counted using a semi-automated Matlab program [27].

#### Recordings of isometric force of muscles and motor units

**Fig 4:**
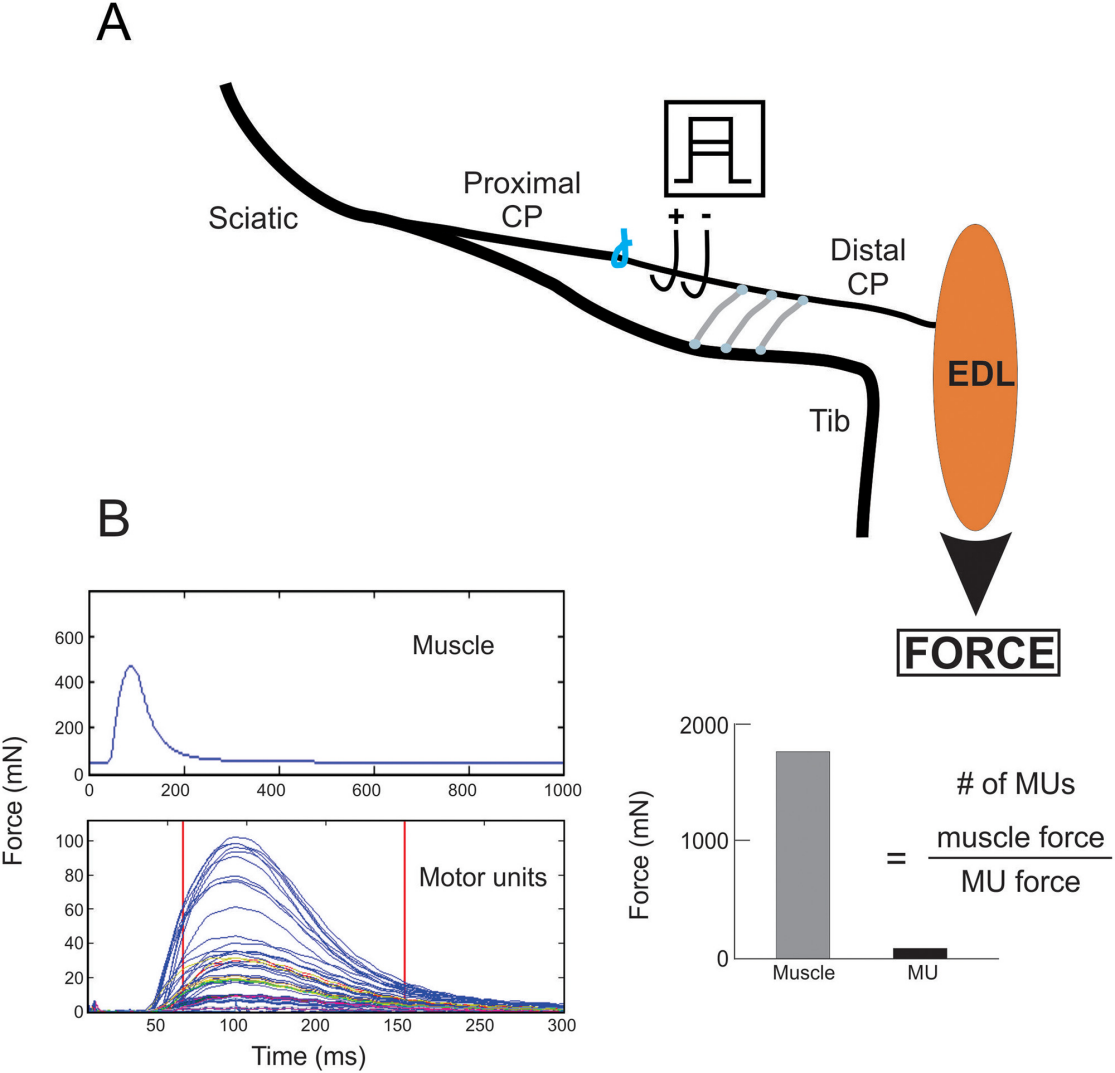
*In vivo* recording of muscle isometric contractile forces to determine numbers of reinnervated motor units. A. In the second set of experiments (Repair, Table 1), bipolar electrodes placed on the regenerated common peroneal (CP) nerve were used to electrically stimulate the CP nerve supramaximally to evoke extensor digitorum longus (EDL) muscle twitch (B) and tetanic isometric contractions or to stimulate the CP nerve incrementally in order to evoke all-or-none increments in twitch force. Motor unit (MU) number was calculated as the ratio of the muscle and average MU twitch forces—motor unit number estimation (MUNE).

In 12 rats, the right experimental hindlimb was prepared for recording of isometric contractions of the extensor digitorum longus (EDL) muscle under deep isofluorane anesthesia and at 37°C, five months after surgical coaptation of the CP nerve stumps with and without placement of three autologous CP cross-bridges (Fig 4 and Table 1: Exp. 2A). The femoral condyles were anchored in a custom-designed force measurement apparatus (Red Rock Inc, St. Louis, MO) and the EDL muscle tendon isolated and attached to a 2 N thin film load cell (S100, Strain Measurement Devices Inc., Meriden, CT) to record isometric force. The CP nerve distal to the site of coaptation was freed for electrical stimulation to 1) evoke EDL muscle twitch (Fig 4A) and tetanic contractions in response to supramaximal (2X threshold) bipolar stimuli, and 2) record motor unit twitch contractions in response to progressive increases of stimulus voltage from 0 V upwards with 100 μs long biphasic stimuli delivered at 0.5 Hz (Fig 4B). Motor units (MUs) were recruited in all-or-none steps of voltage to a maximum of 60% of the maximum muscle twitch force. The mean motor unit twitch force was determined by motor unit number estimation (MUNE). MUNE (number of MUs) was computed by the division of the maximal muscle twitch force divided by the mean contractile force of the motor units (Fig 4B), as described in detail previously [28,29]. MUNE provides an estimation of the number of the CP motoneurons that reinnervated the EDL muscle.

#### Statistics

The data are plotted with mean ± standard errors (SEs) as well as individual data points in Fig 3B. Differences between mean values were evaluated using a one-way analysis of variance (ANOVA) followed by a post-hoc Tukey’s multiple comparisons of means (*p≤*0.05).

## Results

### More neurons send axons through larger perineurial windows

Autologous common peroneal (CP) nerve cross-bridges from the left hindlimb were dissected and placed in a side-to-side fashion via perineurial windows that were opened at equal distances within a 10 mm length of the intact donor tibial (TIB) nerve and the recipient denervated CP nerve stump in the right experimental hindlimb (Fig 1). TIB axonal sprouting and/or regeneration through the cross-bridges and into the distal end of the recipient denervated CP nerve stump was/were determined three months later using retrograde dyes to backlabel motor and sensory TIB neurons (Fig 3). The TIB neurons that *regenerated* their axons into the recipient CP nerve stump were backlabelled with the dye applied to the CP nerve stump but *not* with dye applied to the donor TIB nerve. The TIB neurons that *sprouted* their axons on the other hand were backlabelled from *both* the recipient CP nerve stump and the donor TIB nerve (Fig 3A).

It was reported that the size of the perineurial window is an important determinant of how many axons regenerated through a denervated distal nerve stump inserted into the side of an intact donor nerve—an end-to-side coaptation [30–32]. We first addressed the question of how the size of the perineurial windows impacts on the crossing of donor axons into a recipient denervated nerve stump via side-to-side cross-bridges, an important question that had not been addressed previously (Exp. 1A in Table 1). When small perineurial windows of ~125 μm radius were cut in the donor TIB nerve and the recipient denervated CP nerve stump to accommodate the autologous CP nerve cross-bridges, few and variable numbers of donor TIB motoneurons (<1%) were backlabelled from the recipient denervated CP nerve stump: mean (±SE) numbers of labeled TIB motoneurons that sent axons into the CP nerve stump proximal and distal to the bridges were 8.3 ± 3.3 and 4.5 ± 1.8 (n = 7) respectively for 3 bridges, 0.5 ± 0.6 and 14.2 ± 7.9 (n = 5) respectively for 5 bridges, and 2.1 ± 1.2 and 11.0 ± 9.4 (n = 8), respectively for 7 bridges.

**Fig 5:**
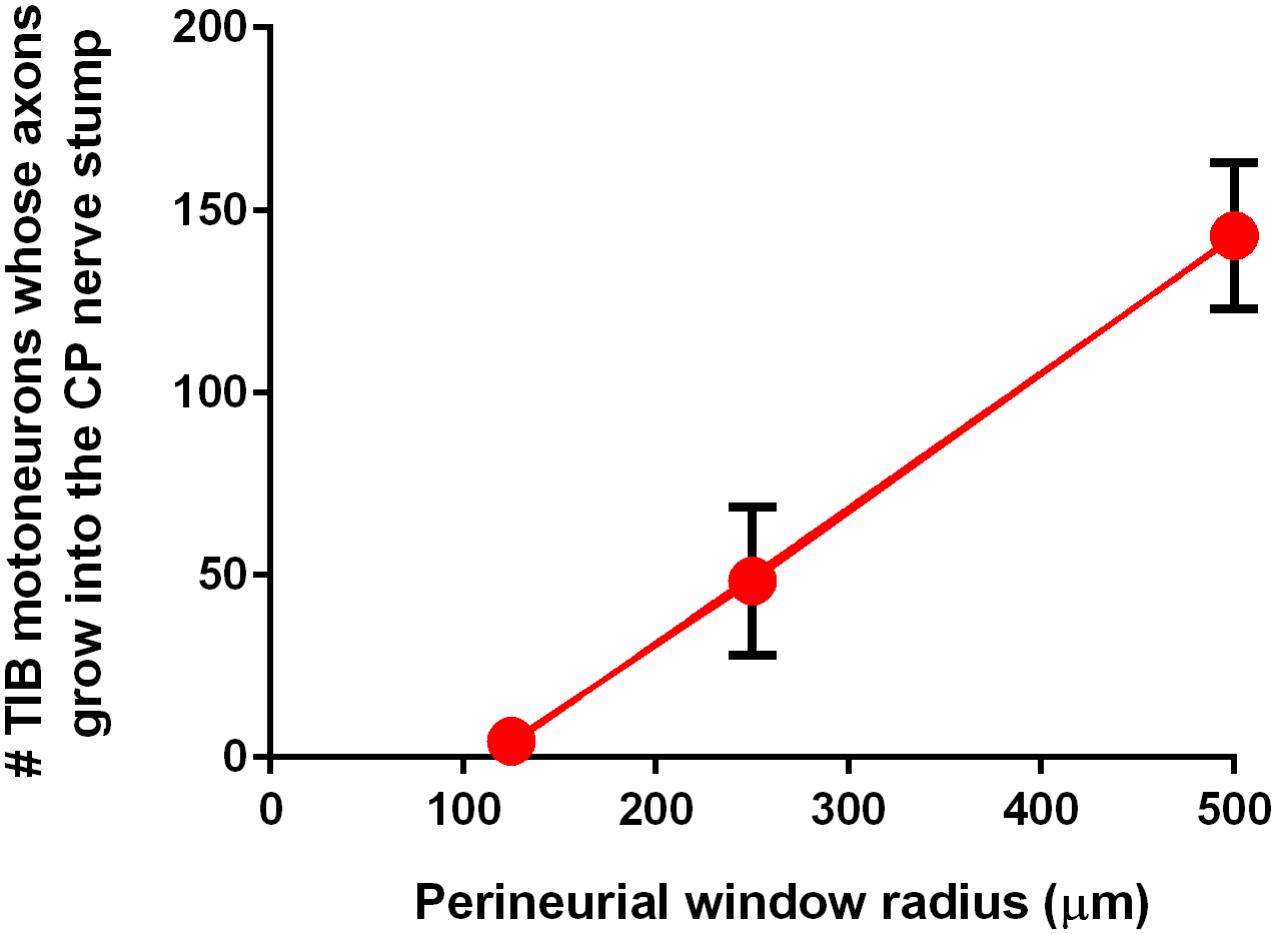
Linear relationship between the numbers of donor neurons sending their axons across 3 side-to-side cross-bridges and the size of the perineurial windows inserted in the nerves. Mean (±SE) numbers of tibial (TIB) motoneurons whose axons crossed 3 side-to-side cross-bridges and were backlabelled with dyes applied distal to the cross-bridges. Note that the error bars that are smaller than the size of the circular marker are not visible.

When wider perineurial windows were opened, significantly more donor TIB motoneurons sent their axons through three cross-bridges into the recipient denervated CP nerve stump, the mean number increasing as a linear function of the radius of the window (Fig 5). Despite considerable scatter in the numbers of TIB motoneurons that regenerating axons into the recipient CP nerve stump through one and three windows with a radius of 250 μm (Fig 3B), the mean numbers of motoneurons sending their axons through these perineurial windows were significantly greater than through smaller windows of 125 μm radius. The numbers increased further when the radius of the perineurial windows was doubled to 500 μm (Fig 5). These data demonstrate that the numbers of neurons that regenerate their axons through cross-bridges between a donor nerve and a recipient denervated nerve stump depend on the size of the perineurial windows that are opened to place the cross-bridges.

Note in Fig 3B that the number of TIB neurons that regenerated their axons through a single cross-bridge was small relative to the numbers that regenerated their axons through three cross-bridges. Moreover, TIB neurons regenerated their axons into the recipient denervated CP nerve stumps in only 50% of the rats in which a single cross-bridge was placed (Fig 3C). Relative to the number of TIB neurons regenerating their axons through one and three cross-bridges into the recipient denervated CP nerve stump, there was only ~0.16% of total TIB motoneuron pool (737 ± 16) that sprouted axons through a single bridge into the denervated CP distal nerve stump (Fig 3B). The mean number (11.7 ± 3.5) of TIB neurons that sprouted axons increased 10-fold when three cross-bridges were placed but the proportion of 1.6% donor motoneurons that sprouted their axons was very small. These findings indicated that increasing the number of bridges was effective in promoting the regeneration and to a much lesser extent, the sprouting of donor axons through the bridges and into the recipient denervated nerve (Exp. 1A in Table 1).

### Donor motor and sensory neurons send axons across side-to-side cross-bridges

**Fig 6:**
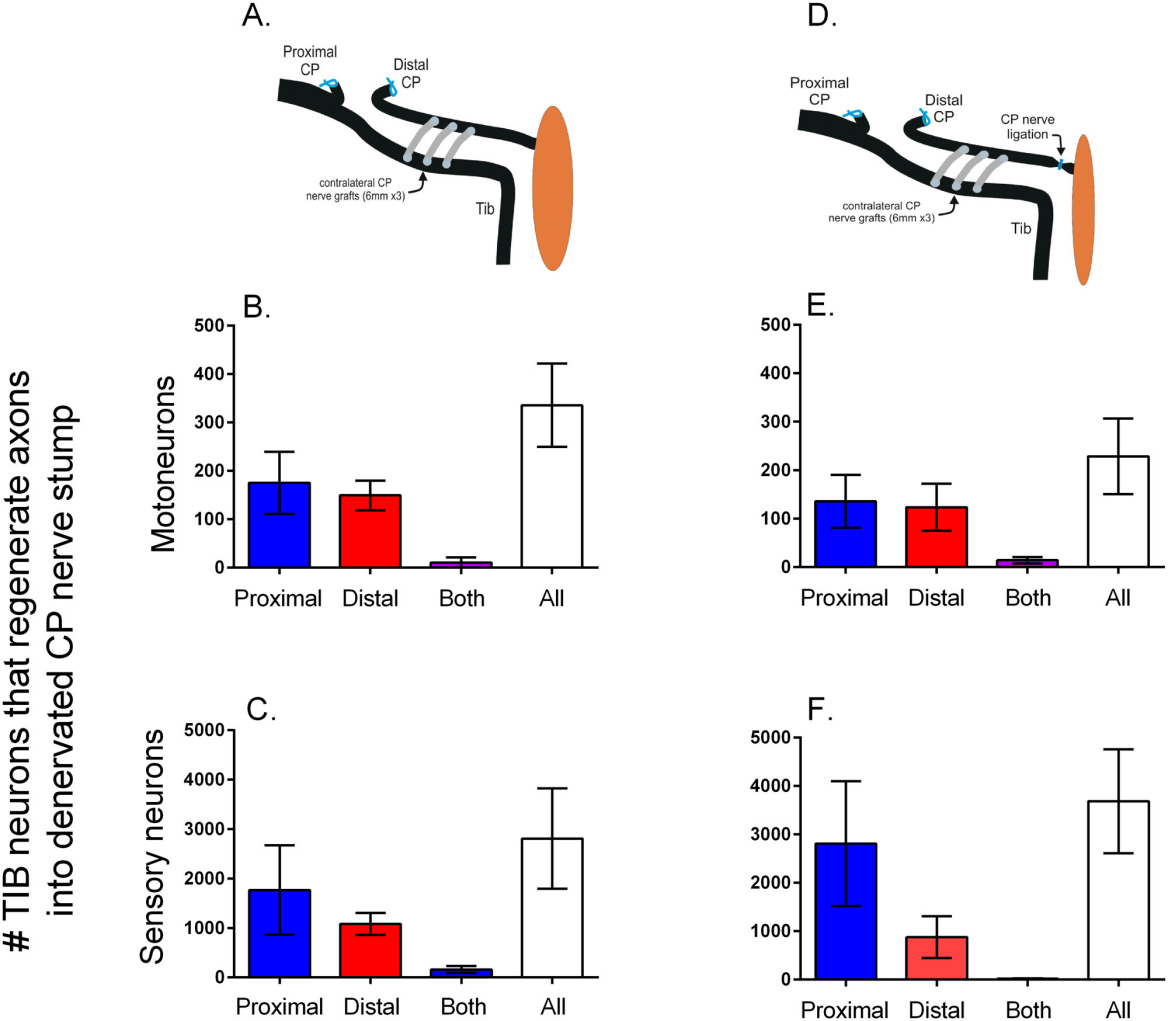
Donor neurons sending axons through cross-bridges proceed equally well in both proximal and distal directions in the recipient denervated nerve stump regardless of whether or not the nerve stump is in contact with denervated targets. In the first set of experiments (No Repair: Table 1), A. 3 cross-bridges of 3.2 mm length and 500 μm radius were placed between donor tibial (TIB) nerve and the recipient denervated common peroneal (CP) nerve stump. The mean numbers (±SE) of both the motor (B) and sensory (C) TIB neurons that regenerated axons into the recipient CP nerve stump proximal and distal to the bridges was the same with very few neurons sending axons in both directions. D. The ligation of the denervated CP nerve stump to isolate the stump from possible neurotrophic effects of the denervated targets, did not significantly affect the numbers of E. motor and F. sensory neurons that sent their axons in both directions, negating possible neurotrophic effects. Each of two retrograde fluorescent dyes were applied either proximal or distal to the cross-bridges to backlabel the TIB neurons as shown in Fig 2A.

In order to determine the direction of growth of donor TIB axons through three CP nerve autografts and into the recipient denervated CP nerve stump (Exp. 1B in Table 1), two different dyes were applied to the CP nerve stump 5 mm proximal and distal to three 3.2 mm long CP cross-bridges placed between the ~500 μm perineurial windows in the two nerves (Fig 2A). Donor TIB motoneurons regenerated their axons through the cross-bridges and equally well in both directions within the recipient CP nerve stump; very few neurons regenerated their axons in both directions (neurons co-labelled from dyes applied proximal and distal to the bridges) (Fig 6A and 6B). The number of TIB dorsal root ganglion (DRG) sensory neurons that regenerated their axons into the recipient denervated CP nerve stump was also not significantly different either side of the three bridges (Fig 6C). The sum of the TIB neurons that regenerated axons into the recipient denervated CP nerve stump was 335 ± 86 for the motoneurons and 2809 ± 1018 for the DRG sensory neurons (Fig 6B and 6C). These numbers correspond to ~32% and ~37% of the total number of TIB motor and sensory neurons of 900 and 8900, respectively [33].

It is unlikely that a ~35% reduction in the numbers of TIB motoneurons that retained intact axons within the donor TIB nerve would result in substantial denervation of the extensor muscles, including the triceps surae muscles that are innervated by these motoneurons. This is because the 4-5-fold maximal enlargement of functional motor units by axonal sprouting is sufficient to fully reinnervate these muscles [34]. Correspondingly, the average mass of the gastrocnemius muscles in the right experimental hindlimb was 1531.6 ± 53 mg. This mass was not significantly different from the average mass of the muscles of 1403.4 ± 70 mg in the left control hindlimb in which the TIB nerve was intact but CP distal nerve stump was removed to construct the autologous CP cross-bridges that were placed in the experimental right hindlimb (see Methods).

**Fig 7:**
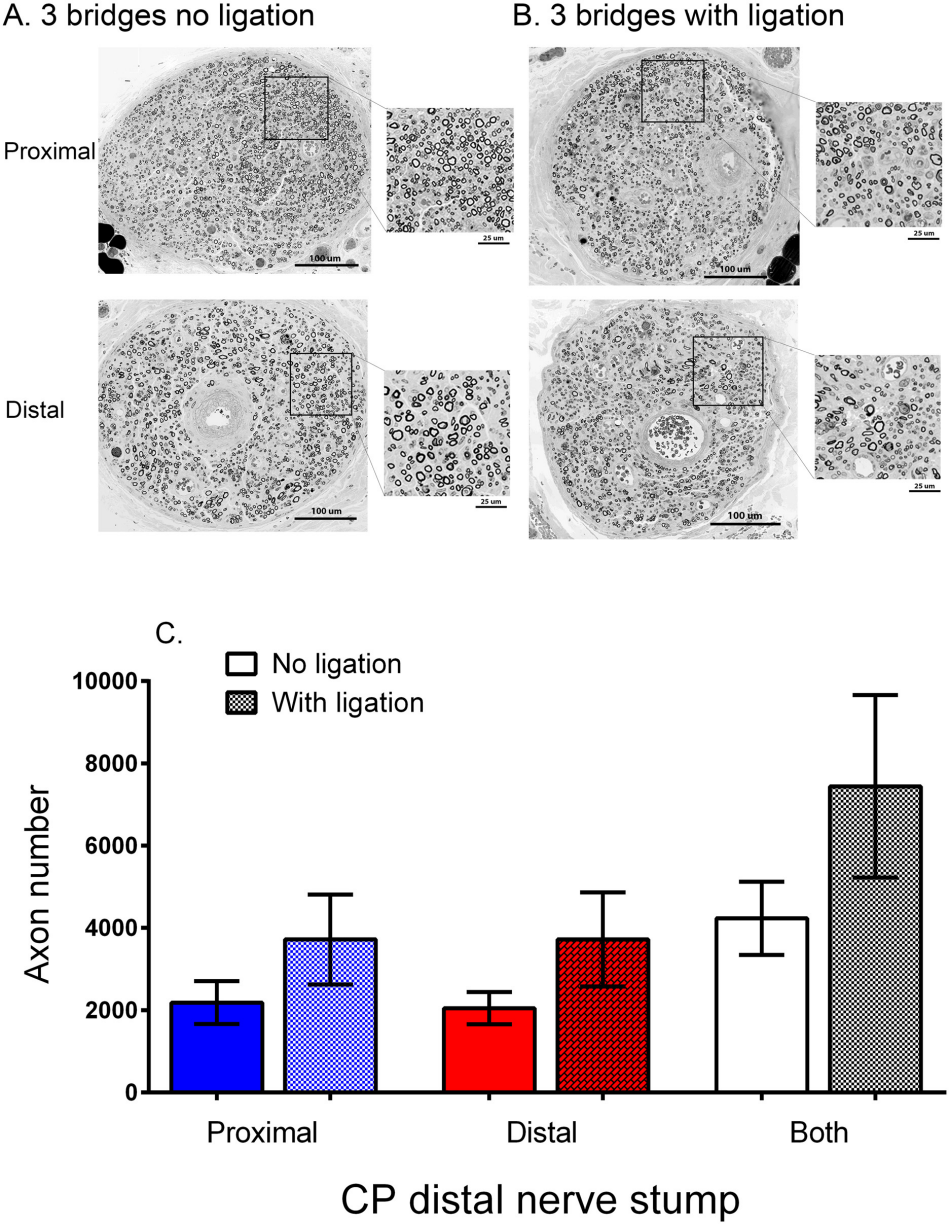
Donor axons regenerate and become myelinated equally well either side of cross-bridges placed between the donor tibial (TIB) nerve and the recipient denervated common peroneal (CP) whether or nor the distal nerve stump is isolated from denervated targets. A. Cross-sections of regenerated TIB axons within the recipient denervated CP nerve stump, proximal and distal to the insertion of 3 cross-bridges between the nerves 3 months previously (Experiment #1, No Repair: Table 1). TIB axons that grew within the CP nerve stump distal to the cross-bridges tended to be larger than B. those in the CP nerve stump that was ligated, consistent with findings that regenerated axons recover size only after making functional contacts. C. Mean (±SE) TIB axon numbers in the recipient denervated CP nerve stump were the same proximal and distal to 3 cross-bridges, whether or not the CP nerve stump was isolated from denervated targets. This negates possible neurotrophic target influences on donor TIB nerves growing into a recipient denervated CP nerve stump.

Myelinated donor TIB axons were found throughout the cross-section of the recipient CP nerve stumps both proximal and distal to the three cross-bridges placed between the donor TIB nerve and the recipient denervated CP nerve stump (Fig 7A). Intact perineurium surrounded single nerve fascicles; there appeared to be more large-diameter axons in the CP nerve stump distal to the bridges three months after placing the cross-bridges, a period of time when some cross-reinnervation of the flexor muscles by the TIB nerves occurs (unpublished observations). The number of donor TIB axons in the denervated CP nerve stump was the same proximal and distal to the cross-bridges (Fig 7C). The large variability in these numbers was consistent with the variability in the numbers of motor and sensory neurons that regenerated their axons into the CP nerve stumps, proximal and distal to the bridges (cf Fig 6B and 6C).

Ligation of the CP nerve stump distal to the last cross-bridge did not affect how many motor and sensory TIB neurons regenerated their axons proximally and/or distally into the recipient denervated CP nerve stump (Fig 6D, 6E and 6F; Exp. 1B in Table 1). The spatial distribution and the numbers of regenerated TIB nerve fibers was also unaffected both proximal and distal to the three cross-bridges (Fig 7B and 7C). These findings discounted possible neurotrophic effects of denervated targets on the direction taken by the regenerated TIB axons within the recipient denervated CP distal nerve stump. Even though there was a trend for the total *number of TIB axons* that grew into the recipient CP nerve stump to be higher than when the CP nerve stump was not ligated distally, this trend was not statistically significant (Fig 7C). The *size* of the TIB axons in the ligated distal CP nerve stump was noticeably smaller as compared to the size when the distal nerve stump was not ligated (cf Fig 7B and 7C) consistent with findings that axon diameter of regenerated axons recovers only after functional contacts are made [35].

### Removing the perineurium

**Fig 8:**
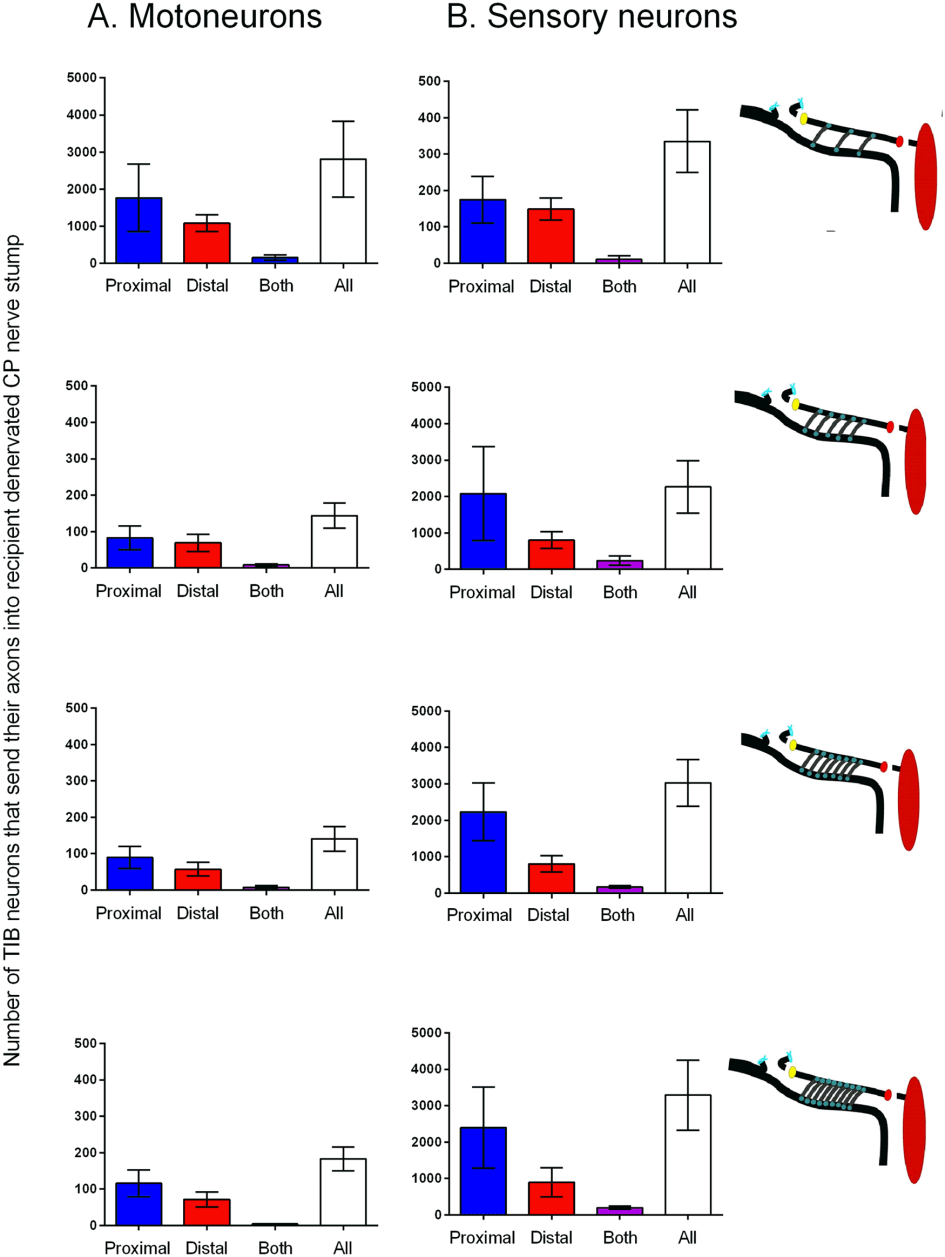
Donor tibial (TIB) neurons grew their axons across cross-bridges into a recipient denervated common peroneal (CP) distal nerve stump where they proceeded to grow both proximal and distal to the cross-bridges. In Experiment #1, No Repair: Table 1, motor and sensory neurons were backlabelled with two fluorescent dyes applied proximal and distal to the cross-bridges (see Fig 2A). The numbers (±SE) of TIB motoneurons and sensory neurons that regenerated their axons into the denervated CP distal nerve stump both proximal and distal to 3, 5, 7 and 9 cross-bridges (as illustrated) were not significantly different. Very few neurons regenerated axons both proximal and distal to the cross-bridges as shown by the few neurons that were double-labelled with two retrograde dyes.

**Fig 9:**
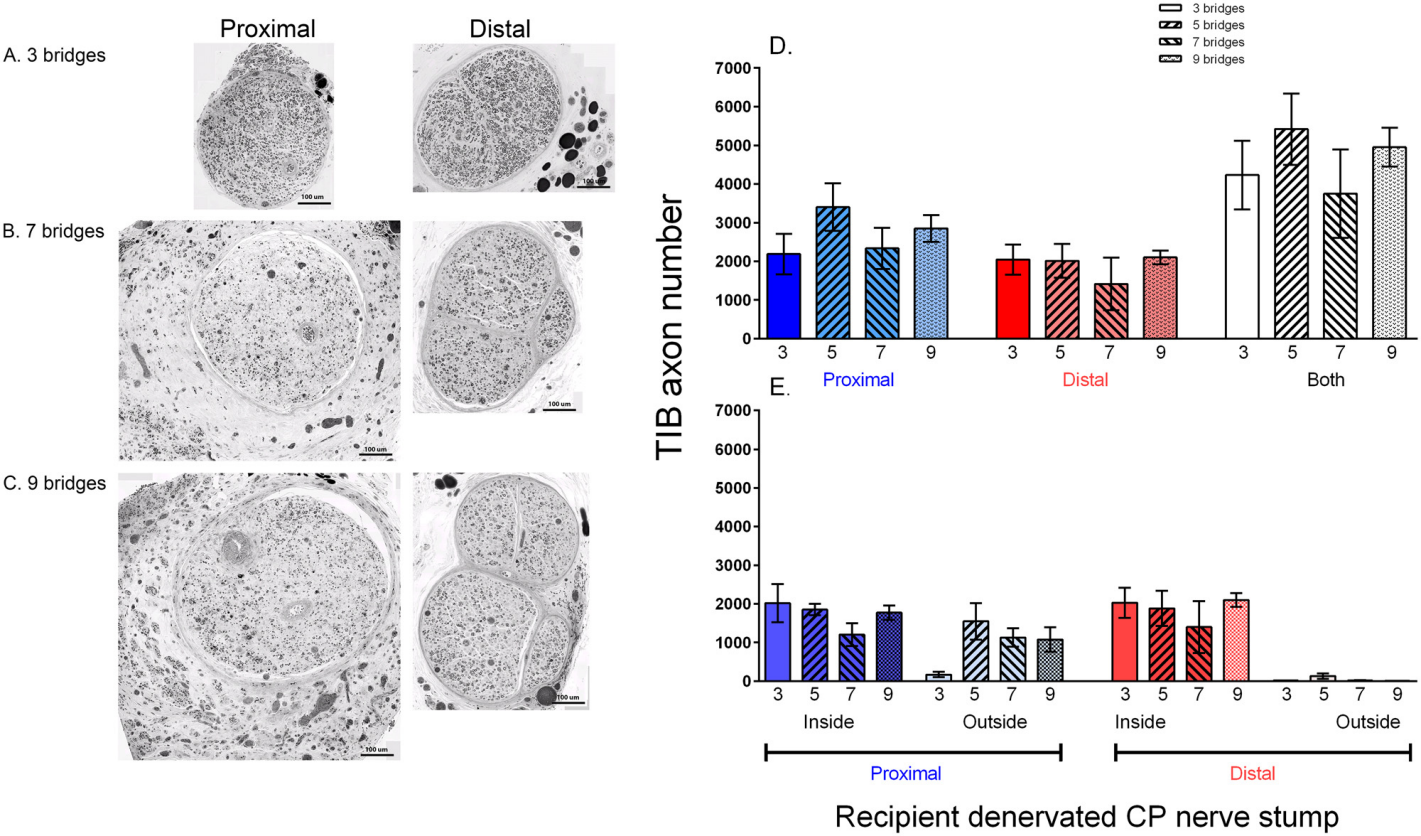
Regenerated tibial (TIB) axons lie outside as well as inside perineurium in the recipient common peroneal (CP) nerve stump proximal to the 10 mm length of the donor TIB nerve and the recipient CP nerve stump where the perineurium was stripped to place 5, 7, or 9 cross-bridges between the donor TIB nerve and recipient CP nerve stump. Cross-sections of the recipient denervated CP nerve either side of A. 3, B. 7 and C. 9 cross-bridges that were placed between the donor TIB nerve and recipient denervated CP nerve stump (Experiment #1, No Repair: Table 1). Regenerated and myelinated TIB axons distal to the cross-bridges were multi-fasciculated when the perineurium was removed to place 5–9 cross-bridges (B,C). D. The means (±SE) of the numbers of TIB axons that regenerated proximal and distal to the cross-bridges were not statistically different irrespective of the number of cross-bridges that were placed whilst E. the same numbers (means ± SE) of TIB axons regenerated within and outside of the perineurium of the CP nerve stump proximal but not distal to 5, 7 and 9 cross-bridges.

The connective tissue sheath of the perineurium of the donor TIB nerve and the recipient denervated CP nerve stump was intact over the 10 mm distance of insertion of three cross-bridges but it was removed to insert five, seven, and nine cross-bridges between the nerves (Fig 1; Exp. 1C in Table 1). The numbers of motor and sensory neurons that regenerating their axons through more than three cross-bridges did not increase, their numbers being the same as when three cross-bridges were placed between perineurial windows (Fig 8). The numbers of donor TIB axons that grew across the bridges into the CP nerve stump proximal and distal to the bridges were also the same, irrespective of the number of bridges (Fig 9D). However, myelinated TIB axons were found in equal numbers inside *and* outside of the original CP nerve fascicle proximal to the placement of more than three cross-bridges after stripping the perineurium (Fig 9B, 9C and 9E). This was in contrast to the containment of the donor TIB axons within the single fascicle of the recipient CP nerve stump when they regenerated their axons through perineurial windows (Fig 9A). Yet, the donor TIB axons were contained inside of the perineurium distal to the five, seven and nine cross-bridges in the CP nerve stump (Fig 9B, 9C, and 9E). Typically there were two or more fascicles in the distal CP nerve stump unlike the normal CP axon distribution within a single fascicle in the popliteal fossa.

### Improved nerve regeneration through ‘protected’ nerve stumps after delayed repair

**Fig 10:**
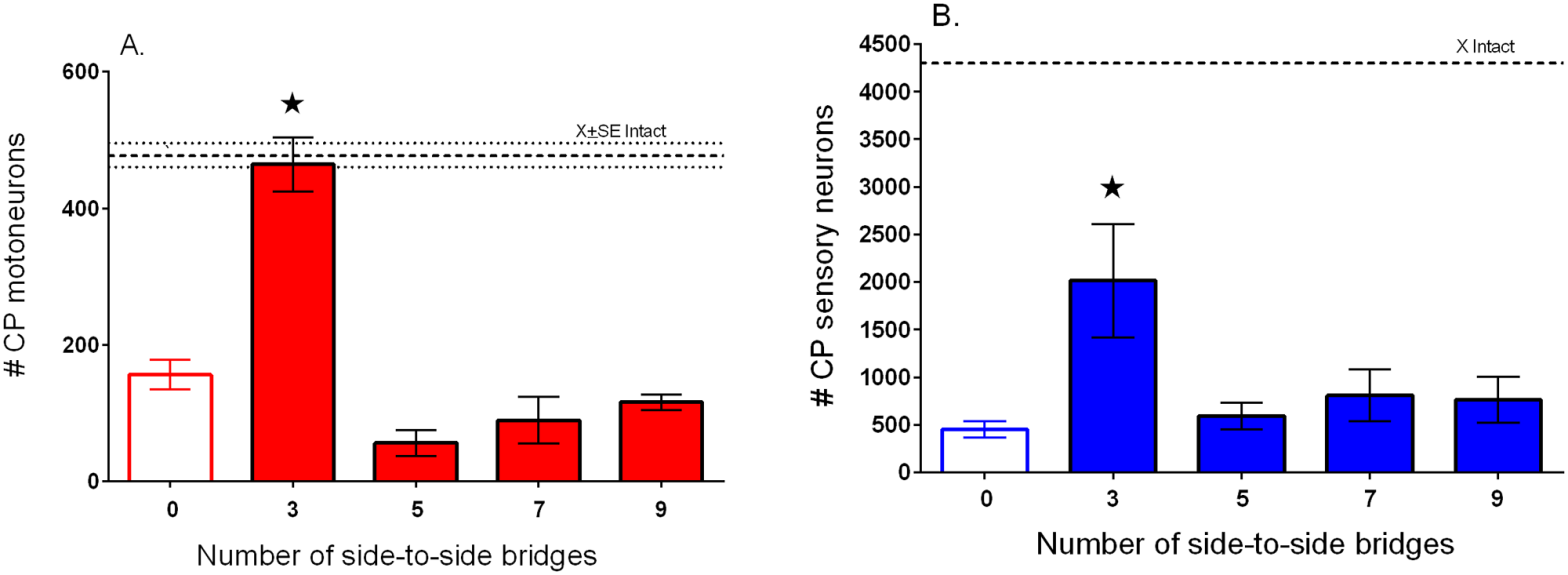
Three but not more cross-bridges placed between a donor tibial (TIB) nerve and a recipient denervated common peroneal (CP) nerve enhance CP motor and sensory nerve regeneration after a 3 month delayed repair of the transected CP nerve. Mean (±SE) numbers of CP A. motor and B. sensory neurons that regenerated their axons through the chronically denervated CP distal nerve stump as a function of the number of side-to-side cross-bridges placed between the donor TIB nerve and the recipient CP nerve stump prior to delayed surgical repair (coaptation) of the transected CP nerve.

We had previously reported that insertion of three cross-bridges through perineurial windows with a radius of 250 μm, in the donor TIB nerve and the recipient denervated CP distal nerve stump three months before a delayed CP nerve coaptation, increased the number of CP motoneurons that regenerated their axons by a factor of 1.7-fold [12]. In this study, we inserted three, five, seven, and nine cross-bridges. The three bridges were placed between perineurial windows of twice the radius, namely 500 μm, and the perineurium was stripped over the 10 mm distance of the cross-bridge insertion when more than three cross-bridges were placed (Exp. 2A and B in Table 1). *All* the chronically axotomized CP motoneurons regenerated their axons through the three month chronically denervated CP nerve stump when they were ‘protected’ by three cross-bridges as compared to only ~33% of the motoneurons regenerating their axons through the nerve stump that was not ‘protected’ by cross-bridges placed between the donor TIB nerve and the denervated CP distal stump over the three month period prior to CP coaptation (Fig 10A). The chronic axotomy of the neurons and the chronic denervation of the distal nerve stumps three months prior to surgical repair account for the poor motor regeneration after delayed nerve repair [3, 7,8,25,36–38]. The same 3-fold increase in the number of chronically axotomized DRG sensory neurons that regenerated their axons after the three month delayed nerve repair of the CP nerve was also seen when three cross-bridges were placed to ‘protect’ the chronically denervated CP nerve stump (Fig 10B). The chronic axotomy of the sensory neurons reduced sensory nerve regeneration to 11%, a new finding that reflects the greater susceptibility of sensory neurons to axotomy [39–40]. Note that the cross-bridges were cut at the time of backlabelling the CP nerve distal to the cross-bridges so as to backlabel only the CP neurons that had regenerated their axons after the delayed nerve repair and *not* the donor TIB neurons whose axons crossed into the denervated CP distal nerve stumps via the cross-bridges (Fig 2B and 2C).

In summary, these data demonstrate that the protective effect of donor TIB axons on chronically denervated Schwann cells facilitates delayed nerve regeneration through the Schwann cells and requires that the axons are contained within the perineurium of the recipient denervated CP nerve stumps (Fig 9).

### Donor axon 'protection' promotes reinnervation of denervated muscles

**Fig 11:**
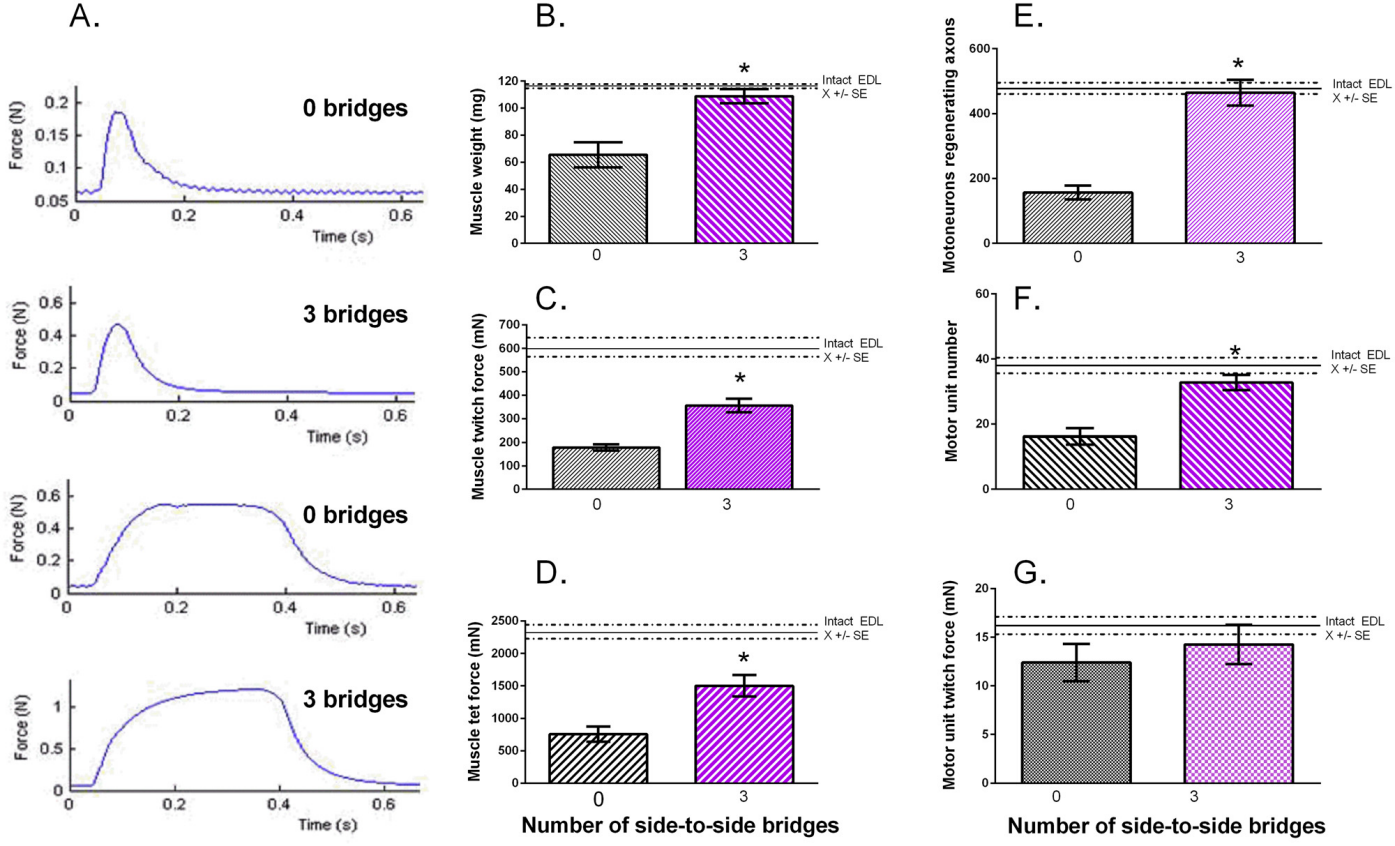
Donor tibial (TIB) axons 'protect' recipient denervated common peroneal (CP) nerve stumps to promote regeneration of CP axons and reinnervation of denervated muscles within 5 months after a 3 month delayed CP nerve repair. A. Examples of maximal twitch and tetanic contractions of extensor digitorum longus (EDL) muscle. These were recorded in response to 0.5 Hz and 100 Hz repetitive CP nerve stimulation at 2X threshold showing increased contractile forces when 3 as compared to 0 bridges were placed between donor TIB nerve and the recipient CP nerve stump 3 months prior to the delayed CP nerve repair (coaptation). (Note the smaller Y-axes for 0 as compared to 3 cross-bridges). The mean (±SE) values of muscle B. wet weight, C. twitch and D. tetanic forces and of the numbers of E. CP motoneurons that regenerated their axons and F. motor units (measured as shown in Fig 4) were significantly increased when 3 cross-bridges were placed as compared to no cross-bridges placed. G. The mean (±SE) values of motor unit twitch forces were not significantly different, the return of all the regenerating motor axons reinnervating all the denervated muscle fibers.

In order to determine whether muscle reinnervation is improved when CP axons regenerate through distal nerve stumps that were ‘protected’ by three cross-bridges, muscle isometric forces and the twitch forces of the muscle fibers innervated by single motor nerves, were recorded (Exp. 2A in Table 1). Typical twitch and tetanic isometric contractile forces developed by reinnervated extensor digitorum longus (EDL) muscles were evoked by electrical stimulation of the CP nerve distal to the placement of the cross-bridges. The electrical pulses were set at 2X threshold to evoke twitch and tetanic contractions in response a single pulse and to repetitive pulses at 100 Hz, respectively. The contractile forces were significantly greater in amplitude when the chronically denervated distal CP nerve stumps were ‘protected’ by three cross-bridges as compared to when no bridges were placed prior to the three month delayed CP nerve coaptation (Fig 11A; note that the force ranges on the Y-axis are three times greater when three cross-bridges as compared to no bridges, were placed). Mean contractile forces were significantly larger although they did not reach preoperative levels within the five months of nerve regeneration but the wet weight of the muscles recovered completely when the chronically denervated CP nerve stump was ‘protected’ (Fig 11B, 11C, and 11D). Of note, development of both twitch and tetanic contractions were slower in the former as compared to the latter conditions.

The calculated number of reinnervated motor units [the ratio of maximal muscle and mean single motor unit twitch forces (Fig 4)] reached normal levels (Fig 11F), consistent with *all* the motoneurons regenerating axons through the CP nerve stump that was ‘protected’ by three cross-bridges after delayed CP nerve coaptation (Fig 11E). The mean twitch contractile forces of the reinnervated MUs were the same whether zero or three cross-bridges were placed (Fig 11G). Hence, the recording of muscle and motor unit forces validated the counts of the motoneurons that regenerated their axons after delayed nerve repair when the chronically denervated CP nerve stump was ‘protected’ by donor TIB axons.

## Discussion

The regenerative capacity of injured neurons declines with time and distance from their denervated targets as they regenerate their axons but fail to reach their targets (chronic axotomy) and the Schwann cells in the distal nerve stumps remain chronically denervated [41]. We demonstrated here that regenerative capacity is restored by encouraging donor axons from an otherwise intact donor nerve to grow across side-to-side autologous cross-bridges into a recipient denervated distal nerve stump prior to the nerve regeneration of the original axotomized neurons. These finding have clear clinical implications for application because first, both motor and sensory neurons demonstrate equal capacity to grow axons from a donor nerve into a recipient denervated nerve stump and second, the neurons send their axons both proximal and distal to the cross-bridges. Surgical repair of injured nerves may be delayed or, even after immediate nerve repair of brachial plexus injuries for example, regenerating nerves typically grow over long and therefore challenging distances. Under these conditions, the relatively simple surgical procedure of placement of side-to-side bridges between an adjacent intact donor nerve and a recipient denervated nerve stump would encourage the occupation of the chronically denervated nerve stumps and their ‘protection’ with and by donor axons during the lengthy period of growth of regenerating axons from the more proximal nerve stumps. This in turn, would enhance the passage of the regenerating axons from the proximal nerve stump and would lead in turn, to improved motor and sensory outcomes in patients.

Studies have indicated that donor axons cross an end-to-side nerve repair (end-to-side neurorrhaphy) whether or not a perineurial window is created [18,19,42]. On the other hand, other studies have suggested that perineurial windows are essential [43,44]. Histomorphological examination of axons in the denervated nerve stump showed that more axons crossed through the perineurial window and into the nerve stump when the window was widened [14,30–32]. We have in this study, examined whether this is the case for two end-to-side neurorrhaphies of autologous nerve grafts placed between a donor nerve and a recipient denervated nerve stump (side-to-side cross-bridges). The size of the perineurial windows was increased systematically to demonstrate that there is a linear relationship between the perineurial window size and the number of neurons contributing axons into the recipient denervated nerve stump (Fig 5): few if any neurons regenerated axons through very small perineurial windows and more donor neurons grew axons reliably through larger ones. Sprouting, as defined by the presence of doubly labeled neurons and was reported after end-to-side neurorrhaphy [21], was a minor contributor of donor axons through the side-to-side cross-bridges to the recipient denervated stump (Fig 3). Most of the donor TIB neurons regenerated their axons across the cross-bridges and into the recipient denervated CP nerve stump as measured and defined by their retrograde labelling from axons within the CP nerve stump and their failure to be backlabelled from the donor TIB nerve 10 mm distal to the last cross-bridge (Fig 3A). Very few donor TIB neurons were doubled-labelled from both the donor TIB nerve and the recipient denervated CP nerve stump distal to the last cross-bridge, these neurons sprouting axons through the cross-bridges and into the recipient denervated CP nerve stump (Fig 3B and 3C). Furthermore, both motor *and* sensory neurons contributed axons equally across the side-to-side bridges (Figs 6 and 8) in contrast to the poor growth of motor axons that was reported across an end-to-side neurorrhaphy [21,45], An average of ~32 and ~37% of the donor motor and sensory neurons regenerated their axons through perineurial windows with a radius of 500 μm that were opened to insert three cross-bridges between the donor tibial (TIB) nerve and the recipient common peroneal (CP) denervated nerve stump. The ~35% reduction in the number of intact TIB axons within the donor TIB nerve would not likely compromise the innervation of the extensor muscles that these axons supply. Motor nerves that retain their contact with their target muscle fibers within partially denervated muscles can sprout axons within the muscles to reinnervate denervated muscle fibers [46]. This sprouting capacity is sufficient so that great remaining intact motor nerves can effectively reinnervate *all* the muscle fibers in partially denervated muscles as long as the partial denervation does not exceed 80–85% [34,47,48]. The mass of the gastrocnemius muscles in the experimental hindlimb in which the cross-bridges were placed between the donor TIB and the denervated recipient CP nerve was the same as in the left control hindlimb. This indicates that all of the extensor muscle fibers were innervated three months after the placement of the cross-bridges. Moreover, as sprouting occurs within weeks [48], the recovery of gastrocnemius muscle weight supports the contention that the sprouting had occurred within the muscles in response to the reduced numbers of TIB axons in the donor nerve.

Donor TIB neurons regenerated their axons across two perineurial windows from the donor nerve, through the cross-bridges, and into the recipient denervated CP nerve stump (Figs 5–9). The Schwann cells within the cross-bridges were denervated, the autologous side-to-side cross-bridges having been constructed from the denervated CP nerve harvested from the contralateral hindlimb (Fig 1). These denervated Schwann cells likely attracted and/or supported the axons growing from the donor nerve because, in their absence, axon outgrowth from a donor nerve fails [49]. Regenerating axons gradually grow across a suture site over a prolonged period of up to twenty eight days in the rat as the axons pass through disorganized arrays of extracellular laminin and other glycoproteins at the site [50,51]. Inhibitory chondroitin sulphate proteoglycans contribute to the lengthy axon outgrowth prior to their degradation [52–59]. The regenerating axons from the donor nerve likely cross perineurial windows over a similar protracted period and, indeed, we have observed this ‘staggered’ regeneration of axons through side-to-side cross-bridges in *Thy-1* transgenic rats [60]. During this period of time, the denervated Schwann cells within the cross-bridges enter into a growth permissive state in which they express many neurotrophic factors and p75 receptors that are permissive for axon growth [61–64]. The denervated Schwann cells will also respond to release of neuregulin, cAMP, and other mitogens from ingrowing axons by cell division [65–67]. Donor axons regenerate into the first (most proximal) cross-bridge before the second and the third such that the numbers of myelinated axons are significantly higher within the first as compared to the third bridge one month after their insertion [60]. It is possible that this proximo-distal course of the donor axons transpired because the perineurial windows were opened along the parallel course of the fibers within the nerves. When more cross-bridges were secured in the nerves whose perineurium was stripped to accommodate them, the numbers of donor axons that crossed into the recipient denervated nerve stump did not increase (Fig 9). These regenerating axons were located equally within and outside of the perineurium of the recipient distal nerve stump proximal to the cross-bridges and within perineurium distal to the bridges (Fig 9). Since all axons had crossed the first three bridges, it is likely that either the axons and/or the indwelling fibroblasts elaborated the perineurium around the axons as the axons passed through the 10 mm length of the CP nerve stump into which the cross-bridges were inserted between the donor TIB nerve and the recipient CP nerve stump. The possibility that donor axons might grow preferentially toward denervated end-organs was discounted in the experiments in which the distal end of the denervated nerve stump was ligated: equal numbers of both motor and sensory neurons sent their axons proximal and distal to the cross-bridges (Figs 6 and 7).

Three months after placing the cross-bridges, the mean number of ~4500 donor axons that occupied the recipient denervated nerve stump (Figs 7 and 9) was ~1.5 times the mean total number of ~3000 parent motor and sensory neurons that regenerated the axons (Figs 6 and 8). The ratio of ~1.5 of donor axons within the recipient nerve stump to neurons that regenerated these axons, is small when compared to the ratio of 5 of the regenerated axons distal to a nerve crush to their parent axons proximal to the crush site [68]. The fewer TIB axons that regenerated into the recipient CP denervated nerve stumps may have resulted from the progressive decline in the numbers of the denervated Schwann cells and/or their progressive atrophy after chronic denervation of the recipient denervated nerve stump. The finding that these axons were myelinated (Fig 7) is consistent with our former findings of the myelination of the few axons that succeeded in regenerating into chronically denervated nerve stumps after a delayed surgical repair [36].

It is likely that donor axons regenerate considerably more rapidly across the second as compared to the first perineurial window between each of the cross-bridges and the recipient denervated nerve stump. From the staggered time course of axon regeneration across a suture site of ~28 days, it is likely that the first axons to regenerate across the 3.2 mm long cross-bridges would reach the second window within days. In the course of the first ten days during which the extracellular matrix becomes aligned, Schwann cells cross into the suture site and are arrayed in the Bands of Bungner that in turn, guide regenerating axons into and through the endoneurial tubes of the denervated distal nerve stump [51]. The pace of axon crossing increases so that all the neurons regenerate their axons across the suture site within three to four weeks [50]. Within the three to four week period of axon regeneration across the first suture site of the cross-bridge insertion into the donor nerve, infiltrating macrophages will clear the axonal debris in the cross-bridges that was generated by the Wallerian nerve degeneration of the axons that were isolatedfrom their neuronal cell bodies [69–78]. As a result, many of the regenerating donor axons in the cross-bridges should grow more rapidly through the second perineurial window and into the recipient denervated nerve stump. A conservative estimate for most of the donor axons to cross into the recipient denervated nerve stump would therefore be in the order of eight weeks with the result that many Schwann cells in the recipient stump would be chronically denervated prior to entry of donor axons. The progressive growth of axons across three bridges would extend this period of time for some of the donor axons with the result that the denervation period may be even longer. It is during this period of chronic denervation of the Schwann cells in the recipient denervated nerve stump that the expression of growth associated genes by the Schwann cells would decline, the neurotrophic factors and their receptors declining exponentially from a peak expression between ten and twenty days [5,6,61,62,79,80]. The exponential decline in the growth permissive state of the Schwann cells parallels the corresponding decline in the regeneration of axons [3,7–8].

The rationale of creating the side-to-side cross-bridges prior to delayed nerve repair was to prolong the permissive state of the Schwann cells by passage of donor axons into the recipient denervated nerve stump. This in turn, was to boost the capacity of the axotomized neurons to regenerate their axons within a chronically denervated nerve stump. The occupation of the distal nerve stump by donor TIB axons was increased by increasing the size of the perineurial windows cut into the donor nerve and into the recipient denervated nerve stump (Fig 5). As a result, three times as many motor and sensory CP neurons regenerated their axons into the CP distal nerve stump after delayed repair when cross-bridges were inserted through perineurial windows with a radius of 500 μm, as compared to the numbers that regenerated their axons when no cross-bridges were inserted to ‘protect’ the chronically denervated CP nerve stump (Fig 10). *All* CP motoneurons regenerated their axons through the ‘protected’ distal nerve stump when three cross-bridges were inserted through perineurial windows of a 500 μm radius as compared to the more limited 1.7-fold increase when ~50 of the TIB motoneurons sent their axons into the recipient denervated CP nerve stump through perineurial windows of half the radius, namely 250 μm (Figs 6 and 9 in [12]). The same increase was seen for the sensory neurons but, the chronic injury having reduced the regenerative capacity of the sensory neurons more drastically than for the motoneurons, the increase was short of all the sensory neurons regenerating their axons after the delayed repair (Fig 10B). The efficacy of side-to-side cross-bridges in promoting sensory nerve regeneration after delayed nerve repair is particularly striking given the more severe effect of axotomy on sensory neurons [39,40] with some ensuing cell death [81], and the lower capacity of the injured sensory neurons as compared to injured motoneurons to regenerate their axons after delayed nerve repair when no side-to-side cross-bridges were placed (Fig 10B). Reinnervated muscles recovered their former weight in concert with the regeneration of all the CP motor nerves through the denervated distal nerve stumps that were ‘protected’ by the cross-bridges (Fig 11B, 11E, and 11F). This was so even though the time span of five months in this study for CP nerve regeneration and muscle reinnervation after delayed nerve coaptation was insufficient for the muscles to fully recover their contractile forces (Fig 11A, 11C, and 11D). Nonetheless, the ‘protection’ significantly improved the muscle contractile forces (Fig 11A, 11C, and 11D).

It is likely the donor axons that grew into the denervated nerve stump released agents such as neuregulin that promote the proliferation of the Schwann cells and their expression of regeneration associated genes. Those axons that made contact with Schwann cells were remyelinated by the Schwann cells (Figs 7 and 9), converting their gene expression from growth permissive to the myelinating phenotype [63,64,79,82–84]. Those that did not contact the Schwann cells likely sustained their expression of the regeneration associated genes, at least in part, via these same agents as well as agents that have not yet been identified. These agents in turn, supported the regeneration of axons through the denervated endoneurial sheaths after the delayed CP nerve coaptation.

In the current study, we allowed the babysitting donor axons to remain within the recipient denervated nerve stump after delayed nerve repair. This was done so as to model the clinical scenario of nerve regeneration of injured neurons being progressively compromised by chronic axotomy and chronic SC denervation over time and distance [3,7,8,41]. We transected the cross-bridges at the time of applying retrograde dyes to the regenerated axons in the CP nerve to ensure that the regenerative success of only the CP neurons, and not that of the donor TIB neurons, was determined (Fig 2). The five to nine bridges placed between the donor nerve and the recipient denervated stump that had been stripped of the perineurium did not offer the `protection`that the three cross-bridges had (Fig 10). This was likely due to insufficient donor axons occupying the endoneurial tubes of the recipient denervated nerve stump with as many axons lying outside as inside of the perineurium (Fig 9). The disrupted distal nerve stump may also have contributed to the poor regenerative success after delayed nerve repair. Under clinical human conditions in which cross-bridges may be placed between larger nerves such as the ulnar and the median nerves in the forearm after brachial plexus nerve injuries for example, it may be prudent to rotate the orientation of the cross-bridges around each of the nerves to allow for adequate ‘protection’ of the denervated Schwann cells in the empty endoneurial tubes.

In summary, our animal experiments systematically investigated the optimal size of perineurial windows between a donor nerve and a recipient denervated nerve stump and revealed that windows whose radii were ~3-fold larger than that of the autologous nerve cross-bridges were optimal for promoting excellent nerve repair after delayed nerve surgery.
